# Editorial for the ‘Reciprocal Links between RNA Metabolism and DNA Damage’ Special Issue: July 2023

**DOI:** 10.3390/genes14081570

**Published:** 2023-08-01

**Authors:** Martin Dutertre

**Affiliations:** 1 Institut Curie, PSL Research University, CNRS UMR 3348, INSERM U1278, 91401 Orsay, France; martin.dutertre@curie.fr; 2 RNA Biology, Signaling and Cancer Lab, Université Paris-Saclay, CNRS UMR 3348, 91401 Orsay, France; 3 Équipe Labellisée Ligue Contre le Cancer, 91401 Orsay, France

Two central parts of molecular biology are the control of genome integrity and genome expression. The study of genome integrity has largely relied on detailed analyses of the fundamental processes of DNA replication, recombination, and repair, of their links with each other and with the cell cycle, and of signaling from DNA lesions to effectors involved in these and other processes, globally referred to as the DNA damage response (DDR). Meanwhile, the study of eukaryotic genome expression has revealed that a large part of it (not just genes) is transcribed into a multitude of RNAs; that all the steps of gene expression (from transcription to translation and RNA decay) are regulated; and that multiple steps occur cotranscriptionally, including pre-mRNA degradation, maturation by (alternative) splicing and polyadenylation, subtle modifications like methylation, and release from chromatin.

There are reciprocal links between the control of genome integrity and expression. Firstly, DNA damage widely affects RNA metabolism, and genes controlling genome integrity are coordinately and specifically regulated at multiple levels of RNA synthesis and processing [1,2,3,4]. Secondly, the processes of DNA replication and repair on one hand, and the processes of transcription and cotranscriptional RNA processing on the other hand, are connected and reciprocally impact each other in multiple ways. For example, a major source of genome instability is conflicts between DNA replication and transcription, and defects of cotranscriptional RNA processing can give rise to replication stress and DNA damage [1,4,5]. Another example is the involvement of transcription, noncoding RNAs, R-loops, and RNA-binding proteins in DNA repair and its control [1,5,6]. Finally, connections between genome integrity and expression are involved in the etiology of neurologic and oncologic diseases, and in cell responses to therapeutic (e.g., anticancer) genotoxic agents [4,5].

This Special Issue, entitled ‘Reciprocal links between RNA metabolism and DNA damage’, contains six review papers and one hypothesis paper that address several fast-growing or emerging topics in this field.

Extensive changes in gene expression, at various transcriptional and post-transcriptional levels, are induced upon DNA damage, and conversely, gene expression programs impact the way cells respond to DNA damage. Pluripotent stem cells (PSCs) have particular properties in terms of DDR and the cell cycle, and the generation of induced PSCs by the forced expression of transcription factors is accompanied by oxidative stress and DNA damage. The review by Chen et al. [7] discusses the role of transcriptional regulators in regulating the DDR and cell cycle in PSCs.

The p53 protein encoded by the *TP53* gene is a well-established DDR factor that acts in part as a transcriptional regulator. As reviewed by Gnanasundram et al. [8], in response to DNA damage, the canonical p53 mRNA is regulated at the levels of stability and translation both quantitatively and qualitatively, with an alternative translation initiation site allowing the production of the p53/47 protein isoform. These regulations are mediated by RNA sequences and structures located in the 5′ and 3′ untranslated regions, and remarkably also in the coding sequence (CDS) of the p53 mRNA. These regulatory RNA elements interact with various RNA-binding proteins, including some proteins that are well known to act in the DDR through interactions with proteins (e.g., MDM2 and MDMX/MDM4) and/or DNA (e.g., Ku70-Ku80).

Like transcription regulation, alternative splicing is widely regulated by DNA damage and, in turn, impacts many DDR genes. In this context, Corcos et al. [9] discuss an emerging link between microsatellite (MS) instability, splicing alterations, and cancer. MS sequences are repetitions of mono- or dinucleotides and are often found in introns, especially in the polypyrimidine tract (PPT) splicing element at the intron 3′-end. In cancers of the microsatellite instability (MSI) class, which carry loss-of-function mutations of genes encoding DNA mismatch repair (MMR) proteins, MS sequences are unstable due to replication errors and defective repair. This can lead to PPT shortening and, in turn, skipping of the downstream exon. This turns out to occur in two genes encoding proteins involved in double-strand break (DSB) DNA repair, namely ATM and MRE11, and the authors hypothesize a feed-forward mechanism involving defective DNA repair and altered splicing (in DDR genes) may play a role in MSI cancer progression.

Besides mediating gene transcription and alternative splicing regulation, RNA polymerase II also has roles in DNA repair. Muñoz et al. [10] discuss its role in the transcription-coupled nucleotide excision repair of bulky DNA lesions, that are induced by some DNA-damaging agents and stall DNA-dependent RNA polymerases. In particular, they discuss the role of RNA polymerase II ubiquitylation and degradation in both transcription regulation and DNA repair, following ultraviolet-C irradiation.

Transcription generates R-loops, which are structures involving a DNA:RNA hybrid and a displaced DNA strand. Khan and Danckwardt [11] discuss how physiological R-loops can exert functions (e.g., in transcription and DSB repair), while unscheduled or accumulating R-loops can disturb DNA replication and favor DNA damage and genome instability. They also discuss the increasing list of proteins—including many RNA processing factors—that bind and/or regulate R-loops, and the mutation and regulation of genes that encode such proteins. Finally, they discuss the association of R-loops with various diseases, such as cancers; highlight the diagnostic potential of R-loops and related proteins; and extensively review R-loop detection techniques. Altogether, this review provides tools, a framework, and an impetus for research on R-loops in disease.

As reviewed by Cargill et al. [12], many RNA helicases—among which about a dozen belong to the DEAD-box family—have been involved in the control of genome stability through the regulation of DDR gene expression, the management of R-loops, and possibly DNA repair, because some of them are found at sites of DNA damage, interact with repair proteins, and/or impact repair.

In addition to RNAs involved in R-loops, several types of noncoding RNAs have been shown to play a role in the DDR through various mechanisms, as reviewed by Shaw and Gullerova [13]. Indeed, within damaged cells, some microRNAs and long noncoding RNAs (lncRNAs) regulate the expression of DDR proteins; some lncRNAs bind repair proteins; and some damage-induced RNAs are generated at DSBs and modulate their repair. In addition, intercellular communication from irradiated cells to neighboring cells (bystander effect) involves microRNAs that travel in extracellular vesicles (called exosomes) and regulate the expression of genes in recipient cells.

Altogether, the articles in this Special Issue reinforce the importance of studying the multifaceted connections between RNA metabolism and DNA damage, both in normal cells and in the context of diseases, and highlight some of the most recent concepts and avenues of research in this growing field.
